# Clinical Efficacy Evaluation of Sirolimus in Congenital Hyperinsulinism

**DOI:** 10.1155/2020/7250406

**Published:** 2020-07-22

**Authors:** Somayyeh Hashemian, Reza Jafarzadeh Esfehani, Siroos Karimdadi, Rahim Vakili, Daniel Zamanfar, Amirhossein Sahebkar

**Affiliations:** ^1^Department of Pediatric Diseases, Faculty of Medicine, Mashhad University of Medical Sciences, Mashhad, Iran; ^2^Department of Medical Genetics, Faculty of Medicine, Mashhad University of Medical Sciences, Mashhad, Iran; ^3^Medical Genetic Research Center, Mashhad University of Medical Sciences, Mashhad, Iran; ^4^Diabetes Research Center, Mazandaran University of Medical Sciences, Mazandaran, Babolsar, Iran; ^5^Halal Research Center of IRI, FDA, Tehran, Iran; ^6^Biotechnology Research Center, Pharmaceutical Technology Institute, Mashhad University of Medical Sciences, Mashhad, Iran; ^7^Neurogenic Inflammation Research Center, Mashhad University of Medical Sciences, Mashhad, Iran; ^8^Polish Mother's Memorial Hospital Research Institute (PMMHRI), Lodz, Poland

## Abstract

**Background:**

Congenital hyperinsulinism (CHI) is a rare and life-threatening genetic disorder. Sirolimus as a mammalian target of rapamycin inhibitor may be helpful in patients with CHI who do not respond well to other treatments including diazoxide and octreotide. However, the safety and efficacy of this therapy are still unclear. This study aimed to evaluate the potential therapeutic effects of sirolimus in CHI patients with mutations in the ABCC8 and KCNJ11 genes.

**Methods:**

During the period of this follow-up study, every child with a confirmed diagnosis of unresponsive CHI underwent genetic evaluation. Among those who had positive genetic testing, six families agreed to participate in this study. The participants were evaluated for ABCC8, KCNJ11, or HNF4*α* gene mutations by polymerase chain reaction (PCR) sequencing. The participants who were unresponsive to diazoxide and octreotide therapy received 0.5 mg/m^2^/d of sirolimus, and the dose was gradually increased until a serum concentration of 5–15 ng/ml was achieved. Then, the participants were followed up for any possible complications.

**Results:**

Among the study participants, only one neonate was completely free of hypoglycemia after one year of follow-up, whereas three others experienced a partial reduction in hypoglycemic episodes over six months. One neonate underwent pancreatectomy despite receiving sirolimus. The oldest participant with a mutation in the ABCC8 gene responded well to sirolimus therapy after surgery and remained asymptomatic for 18 months.

**Conclusion:**

This study suggested that sirolimus therapy needs further evaluation to determine which patients will benefit the most. The genetic basis of CHI may have possible implications for determining the patient's response.

## 1. Introduction

Self-limiting hypoglycemia is a common problem that occurs shortly after birth and is managed by regular feeding or dextrose-containing intravenous fluids. However, persistent hypoglycemia is a major concern as it may imply a serious underlying etiology such as congenital hyperinsulinism (CHI) [1]. CHI is a rare genetic disease with an estimated prevalence of 1 per 50,000 live births [2]. The diagnosis of CHI is usually based on clinical assessments, but the pathological evaluation is able to more clearly confirm the diagnosis and classification. Moreover, careful physical examination focusing on dysmorphic features can differentiate syndromic hyperinsulinism from isolated CHI [2]. Recently, genetic studies have also become available and in many cases, and the antenatal diagnosis has become possible. Alterations in some genes including ABCC8, KCNJ11, and HNF4A have been linked to isolated CHI [2]. The main pathophysiologic feature of isolated CHI is an inappropriate increase in insulin secretion which results in prolonged and permanent hypoglycemia. Prolonged hypoglycemia can cause adverse neurodevelopmental outcomes [3]. Hence, prompt management of this serious congenital disorder is important. Sirolimus which is a mammalian target of rapamycin (mTOR) inhibitor may be helpful in patients who do not respond well to other treatment including diazoxide and octreotide [4]. However, it is known from follow-up reports that sirolimus safety and efficacy have not been widely investigated. There are still some major concerns about the side-effects of this drug. This report presented the results of treatment and short-term follow-up of children who have isolated CHI and received sirolimus.

## 2. Methods

This follow-up study had been approved by the Mashhad University of Medical Sciences Ethics Committee and conducted in the Imam Reza Hospital, Mashhad, Iran (Ethics Committee approval code: IR.mumus.fm.REC.13940376). During a 10-year period starting from the late 2007, every neonate/child who had clinical and laboratory features of persistent hypoglycemia underwent assessment of “critical” sample in the hypoglycemic state (with hypoglycemia defined as a plasma glucose less than 50 mg/dL). Cases who had (1) hyperinsulinemia (defined as the plasma insulin greater than 2 uU/mL) or (2) hypofatty acidemia (plasma free fatty acids less than 1.5 mmol/L) or (3) hypoketonemia (defined as a plasma B-hydroxybutyrate less than 2 mmol/L) or (4) inappropriate glycemic response to 1 mg intravenous glucagon (changes in the plasma glucose greater than 40 mg/dL) were defined to have hyperinsulinism, and [5] treatment with dextrose fluid therapy, diazoxide, and then, octreotide was recruited. Thereafter, every neonate/child with a confirmed diagnosis of CHI underwent genetic testing and follow-up for therapy. In patients with unresponsive CHI (defined as recurrent hypoglycemia despite treatment with 15 mg/kg/d diazoxide after at least five days of therapy, 35 mcg/kg/d octreotide, and 10 mg/kg/min of intravenous dextrose), who had positive genetic testing, six families agreed to participate in this study and filled the informed consent form. Genomic DNA was evaluated for ABCC8, KCNJ11, or HNF4*α* gene mutations by polymerase chain reaction (PCR) sequencing. The parents of neonates with known causative mutations were, then, evaluated for the presence of mutations same as those of their offsprings by PCR-sequencing.

Affected individuals were treated with 0.5 mg/m^2^/d of sirolimus (BIOCON, India) after providing written informed consent. None of these neonates were allergic to sirolimus derivatives, macrolides, or other drugs with similar formulations. Moreover, they did not have any history of immunodeficiency or pulmonary disease. The sirolimus dose was gradually increased until a serum concentration of 5–15 ng/ml was achieved. The initial response was assessed over at least 10 days and follow-up continued for at least 6 months. Accordingly, the dose of dextrose, diazoxide, or octreotide was gradually tapered, and if possible, blood glucose monitoring every 4–6 hours was performed and symptoms of hypoglycemia were assessed carefully. During this period, each patient was followed up focusing complications or adverse events. Development of pulmonary disorders, a decrease in white blood cell counts, or unwillingness to continue taking sirolimus resulted in withdrawal from the study and termination of sirolimus medication. Those cases who responded to sirolimus were followed up for up to three years.

## 3. Results

**Figure 1:**
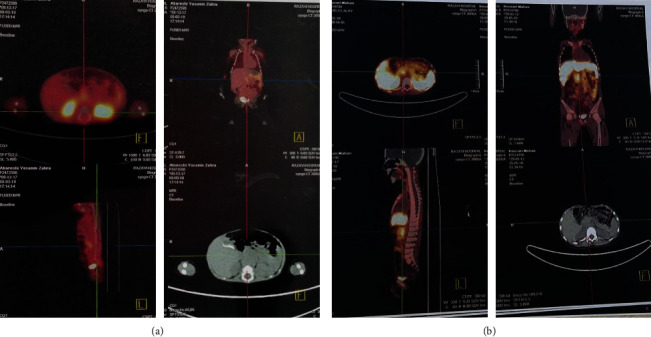
68 Ga-DOTA-TATE PET scan showed a lesion in the head of pancreas in two patients (upper: case 4 and lower: case 6).

During the study period, six out of eight children with unresponsive CHI agreed to enroll in the present study (Table 1). Among the study patients, all of them were termed at the time of delivery, 2 patients were large for gestational age (LGA) (patient number 1 and 5) and one patient was small for gestational age (SGA) (patient number 6). Only the patients 2, 4, and 6 agreed to undergo a DOPA positron emission tomography (PET) scan (Figure 1). The PET scan for our patients showed suspicious lesions in the head of the pancreas. The age of disease onset, as well as sirolimus administration, is demonstrated in Table 2, and the plasma glucose level was evaluated every 4–6 hours. The recommended blood glucose target of >70 mg/dl had a margin of safety for the goal of treatment and reducing neurological complications. Also, severe symptoms of hypoglycemia such as seizures were one of the insufficient responses to therapy [6]. Another criterion for clinical response was the ability to fast for at least 4–6 hours without signs or symptoms of hypoglycemia. Eventually, we followed up our patients in hospital or at home by frequent glucose sampling every 4–6 hours (serum glucose or glucometer) for symptoms of hypoglycemia such as seizures due to poor feeding or forgetting to give medicine by parents. Patients who had their care at home, with evidence of symptomatic hypoglycemia such as seizure, were immediately transferred to the pediatric hospital after receiving medical consultations.

Among the study patients, the first patient received diazoxide and octreotide before undergoing subtotal pancreatectomy for 5 weeks. After surgery, she was treated with sirolimus in 8 weeks of age, due to recurrent episodes of hypoglycemia with glucose and diazoxide therapy. After sirolimus adjuvant therapy, the dose of dextrose was tapered and discontinued after 10 days. Attacks of hypoglycemia decreased and discontinued in the next 2 months, and after 3 months, sirolimus was gradually discontinued and diazoxide therapy continued. She had normal neurodevelopmental growth after one year of follow-up in the absence of hypoglycemia with only diazoxide therapy.

The second patient did not respond to diazoxide and octreotide therapy and underwent proximal pancreatectomy at 4 weeks. However, despite continuing diazoxide and octreotide therapy, she underwent distal pancreatectomy due to persistent episodes of hypoglycemia and seizures at 12 weeks. Because of persistent hypoglycemia, the patient received postoperative diazoxide/octreotide and, then, sirolimus. After 3 weeks, octreotide therapy was discontinued. Hypoglycemic attacks reduced within six months of follow-up, but not all the time. She had no more episodes of seizure, but some attacks of hypoglycemia happened in febrile illness or insufficient feeding state. These two cases had parents with the heterozygous mutations same as those in their offspring.

The third neonate received sirolimus four weeks after starting diazoxide and octreotide therapy. After six months of follow-up, episodes of hypoglycemia were reduced in a normal neurodevelopmental condition.

The fourth infant also received sirolimus after octreotide and diazoxide therapy in the sixth week of age. In the 68Ga-DOTA-TATE PET scan, she had a mild uptake in the region of the head of the pancreas. She underwent subtotal pancreatectomy because of recurrent hypoglycemia six months later. After surgery, she had periodic episodes of hypoglycemia, so diazoxide was started in limited doses again. With adding diazoxide therapy (5 mg/kg/day), episodes of hypoglycemia disappeared. She had two attacks of mild hypoglycemia due to forgetting medication. The fifth neonate had a positive family history (fatal neonatal hypoglycemia affected her sister). Diazoxide, octreotide, and then, sirolimus therapy were initiated at the third week of age. She had normal developmental growth in the follow-up. However, after eight months, she missed close monitoring. The parents of these two patients are heterozygous for the mutation same as that of their offspring.

The last patient was a 3.5-year-old boy with a history of recurrent attacks of seizure since early months of life; however, the patient was mismanaged, and the hypoglycemia was diagnosed by the age of 3.5. Hence, hyperinsulinism was confirmed with confirmatory tests, genetic assay, and imaging evaluations. In the Ga-DOTA-TATE PET scan, there was an avid lesion in the head of the pancreas (Figure 1). He received octreotide and diazoxide therapy because of recurrent episodes of hypoglycemia. Despite medical treatment and Whipple surgery (proximal pancreatectomy), he had some degree of unconsciousness due to hypoglycemic attacks. After starting sirolimus, added to diazoxide, the patient was symptom-free of hypoglycemic attacks for 18 months of follow-up. He had some attacks of hypoglycemia when the dose of sirolimus was tapered, so sirolimus therapy continued in sufficient dose.

## 4. Discussion

Management of CHI is challenging, and possible therapeutic choices are limited. Sirolimus is not widely used in CHI management, and it is considered the last-line therapy. The present report showed possible beneficial effects of sirolimus on persistent hypoglycemia in some CHI patients without notable complications. Among our six patients, one patient responded markedly to this treatment and was asymptomatic for one year of follow-up. Three patients experienced a relative reduction in hypoglycemic events. Another one underwent pancreatectomy after six months of treatment because of recurrent hypoglycemia. The other patient responded well to sirolimus therapy and was asymptomatic for up to 18 months of follow-up. CHI can be subdivided according to clinical presentation, pathological findings, or genetic alterations. Such classification will guide physicians to establish ideal management for their patients. As an example, syndromic CHI, mostly diagnosed according to syndromic features alongside CHI, usually responds well to diazoxide therapy [2]. Our findings demonstrated that therapeutic response may also be accompanied by a genetic mutation in CHI patients. Our six patients had mutations in ABCC8 and KCNJ11 genes [8]. Al-Balwi et al. also evaluated the effect of sirolimus on three infants with CHI [9]. They concluded that, in those who are bearing homozygous mutation, sirolimus would be less effective [9]. However, one of our homozygous patients responded to the therapy without side effects. Even specific mutations in a different zygosity status may be linked to a better response, while it has not been addressed briefly in the literature. Alteration in these genes is responsible for the development of channelopathies of potassium channels in B-cells [2]. Sirolimus therapy was considered an alternative therapy for those who are not responded to diazoxide [10]. In our study, we used treatment protocol for sirolimus same as that used by Senniappan et al. [10]. According to our results, only one neonate responded fully to sirolimus therapy. This mTOR inhibitor can downregulate insulin production and is being used as an off-label drug in some centers. It was suggested that sirolimus can be used as an alternative treatment to surgery in CHI cases in their first year of life [4]. Also, the present study demonstrated that this patient did not require surgery after treatment for up to 6 months. Although the effectiveness of sirolimus as an alternative treatment of CHI has been demonstrated in the literature [10, 11], there are still some clinical side-effects and concerns that should be addressed. The first concern is about the immunosuppressive nature of sirolimus [10]. We monitored our patients' white blood cells count during and after receiving sirolimus for at least one year and did not observe any significant alteration in the WBC count in our patients similar to others [8]. Another concern is the potential hepatotoxicity of sirolimus [11], but no abnormality of liver enzymes was observed in our patients, except in case 2 with mild (less than 2-fold) transient nonsignificant elevation in transaminases. Haliloglu et al. demonstrated that discontinuation or dose reduction of sirolimus leads to normalization of liver function tests within a few days [12]. Regardless of these two main concerns, other side effects including elevation in serum triglyceride, sepsis, anemia, stomatitis, gut dysmotility, and varicella-zoster infection have been reported following sirolimus therapy. Although these side effects were not observed in this study, more attention and extreme caution are needed for all patients regardless of their genetic status [13]. Moreover, in adults, sirolimus therapy is associated with specific side effects. Dastamani et al. reported a case of CHI due to the dominant ABCC8 mutation who was switched from diazoxide to sirolimus because of diazoxide side effects [14]. They reported that the16-year-old patient was involved with cellulitis and diabetes [14].

One of the limitations of the present study is the limited number of participants who agreed to enroll in the present study. Because of limited reports regarding the complications and long outcomes of CHI patients receiving sirolimus, some families did not agree to use this treatment. Another limitation of our present study is the limited follow-up of our patients. The small sample size has prevented us from demonstrating any relationship between genetic variations in ABCC8 or KCNJ11 genes and sirolimus response. We suggest future research with larger sample sizes and longer follow-up periods to investigate the potential therapeutic effects of these drugs.

## 5. Conclusions

Our study demonstrated that sirolimus can be beneficial for some infants with CHI. Our patients have a mutation in both ABCC8 and KCNJ11 genes, and in one neonate who did not undergo surgery (and another one who did not undergo surgery for 8 months follow-up), sirolimus was effective. In an older child (case 6) with ABCC8 mutation after surgery, the sirolimus could successfully control the hypoglycemic episodes for up to 18 months of follow-up. Although we found no side-effects of sirolimus therapy, this needs to be confirmed in future trials with larger patient populations.

## Figures and Tables

**Table 1:** The characteristics of neonates with congenital hyperinsulinemia receiving sirolimus.

	Gender/birth weight	Age of sign/symptoms of hypoglycemia	Plasma glucose level during hypoglycemia (mg/dL)	Insulin level (mciu/ml)	Previous treatment	Previous surgery	Disease-causing variant	ACMG classification^*∗*^
Patient 1	Female/4200 gr	4^th^ day	27	47	Diazoxide and octreotide	Yes (5 weeks)	Homozygous missense variant in ABCC8 gene c.331G > A (p.Gly111Arg)	Uncertain significance

Patient 2	Female/2600 gr	First day	34	63	Diazoxide and octreotide	Yes (4 and 12 weeks) (histologic features: Diffused islet hyperplasia)	Homozygous nonsense variant in ABCC8 gene c.2809C > T (p.Gln937Ter)	Pathogenic

Patient 3	Male/3150 gr	4^th^ day	32	54	Diazoxide and octreotide	No	Heterozygous missense and aberrant splicing in ABCC8 gene c.96C > G and c.2041–21G > A (p.Asn32Lys)	Both uncertain significance

Patient 4	Female/3500 gr	2^nd^ day	36	65	Diazoxide and octreotide	No	Homozygous missense variant in KCNJ11 gene c.370T > A (p.Ser124Thr)	Uncertain significance

Patient 5	Female/4100 gr	First day	28	28	Diazoxide and octreotide	No	Homozygous missense variant in KCNJ11 gene c.362T > G (p.Phe121Cys)	Uncertain significance

Patient 6	Male/2300 gr	From early infancy with attacks of seizure in 3.5 years diagnosed as hyperinsulinism	44	34.6	Diazoxide and octreotide	Yes (4.5 years) (histologic features: Islet hyperplasia)	Heterozygous missense variant in ABCC8 gene c.3446G > A (p.Cys1149Tyr)	Likely pathogenic

ACMG: American College of Medical Genetics. ^*∗*^According to varsome.com (7).

**Table 2:** Assessment of response to treatment with sirolimus and outcome.

Patient no.	Age of starting sirolimus	Dose of sirolimus of starting (mg/m^2^)	Final dose of sirolimus (mg/m^2^)	Response to treatment with sirolimus	Duration of treatment	Side effects	Outcomes and follow-up
1	8 weeks	0.5	0.2 and, then, discontinued	Discontinued dextrose therapy after 10 days	3 months	None	Continue sirolimus therapy for 3 months, no hypoglycemic episodes for one year after tapering sirolimus. Eurodevelopmental growth compatible with the age

2	14 weeks	0.5	1.5	Discontinued dextrose therapy after 15 days and octreotide after 20 days	More than one year until the present	Mild transient elevation of liver aminotransferase levels	Reduced hypoglycemic episodes for 6 months after receiving sirolimus, continued plus diazoxide, and developmental delay

3	4 weeks	0.5	0.7⟶0.4	Discontinued dextrose therapy after 17 days and diazoxide/octreotide after 20–24 days	More than one year (continued until now)	None	Reduced hypoglycemic episodes for 6 months after receiving sirolimus. In 14 months of age, neurodevelopmental growth compatible with the age

4	6 weeks	0.5	1–1.5	Recurrent attacks of hypoglycemia and seizure, discontinued after 6 months	6 months	None	Recurrent hypoglycemia ended up in pancreatectomy after 6 months of receiving sirolimus, after pancreatectomy (focal hyperplasia in histologic findings) diazoxide therapy with minimal dose continued

5	3 weeks	0.5	0.5–0.7	Discontinued dextrose therapy after 25 days, continued diazoxide 15 mg/kg/d and octreotide 10 mcg/kg/d	8 months (discontinued follow-up after 8 months)	None	Reduced hypoglycemic episodes for 6 months after receiving sirolimus (no seizure or poor feeding) adequate developmental growth

6	5 years	0.5	0.5	Continued diazoxide (10–15 mg/kg/d)	More than one year, continued until now	None	After surgery, the hypoglycemia episode was controlled by sirolimus plus diazoxide. No attacks of seizures, reduced hypoglycemia less than 1 time in the week

## Data Availability

Data are available from the corresponding authors upon a reasonable request.
